# Association between vitamin D levels and lower-extremity deep vein thrombosis: a case-control study

**DOI:** 10.1590/1516-3180.2020.0457.R1.04022021

**Published:** 2021-05-28

**Authors:** Ufuk Turan Kursat Korkmaz, Suleyman Ersoy, Ahmet Yuksel, Humeyra Celik, Erhan Renan Ucaroglu, Yusuf Velioglu, Ayhan Cetinkaya, Deniz Demir, Umut Esen, Kemalettin Erdem

**Affiliations:** I BSc, MD. Assistant Professor, Department of Cardiovascular Surgery, School of Medicine, Bolu Abant Izzet Baysal University, Bolu, Turkey.; II BSc, MD. Assistant Professor, Department of Family Medicine, Health Sciences University, Umraniye Training and Research Hospital, Istanbul, Turkey.; III BSc, MD. Associate Professor, Department of Cardiovascular Surgery, School of Medicine, Bolu Abant Izzet Baysal University, Bolu, Turkey.; IV MD, MSc. Specialist Doctor, Department of Physiology, School of Medicine, Bolu Abant Izzet Baysal University, Bolu, Turkey.; V MD, MSc. Specialist Doctor, Department of Cardiovascular Surgery, School of Medicine, Bolu Abant Izzet Baysal University, Bolu, Turkey.; VI BSc, MD. Associate Professor, Department of Cardiovascular Surgery, School of Medicine, Bolu Abant Izzet Baysal University, Bolu, Turkey.; VII PhD. Associate Professor, Department of Physiology, School of Medicine, Bolu Abant Izzet Baysal University, Bolu, Turkey.; VIII BSc, MD. Associate Professor, Department of Cardiovascular Surgery, Health Sciences University, Bursa Yuksek Ihtisas Training and Research Hospital, Bursa, Turkey.; IX BSc, MD. Specialist Doctor, Department of Family Medicine, Basaksehir Cam and Sakura City Hospital, Istanbul, Turkey.; X BSc, MD. Professor, Department of Cardiovascular Surgery, School of Medicine, Bolu Abant Izzet Baysal University, Bolu, Turkey.

**Keywords:** Vitamin D, Venous thrombosis, 25-hydroxyvitamin D3 1-alpha-hydroxylase, Deep vein thrombosis, Deep venous thromboses, 25(OH)D, 25-hydroxyvitamin D3, Thrombosis, deep vein

## Abstract

**BACKGROUND:**

Vitamin D has relationships with pathogenesis and inflammation pathways in many diseases. Its deficiency may make clinicians think not only of supplementation but also of presence of other diseases.

**OBJECTIVE:**

To investigate the relationship between vitamin D levels and deep vein thrombosis (DVT), given that reduced levels are related to increased risk of cardiovascular diseases.

**DESIGN AND SETTING:**

Case-control study conducted in the cardiovascular surgery and family medicine departments of a hospital in Turkey.

**METHODS:**

A total of 280 participants were included: 140 each in the DVT and control groups. Basic clinical characteristics, comorbidities and serum 25-hydroxyvitamin D (25(OH)D) levels were recorded and then compared between the groups. Serum 25(OH)D levels were also evaluated separately in three subgroups (sufficient, insufficient and deficient).

**RESULTS:**

Serum 25(OH)D levels were significantly lower in the DVT group than in the controls (P < 0.001). Females in the DVT group had lower 25(OH)D levels than those in the control group (P = 0.002). Nonetheless, the median 25(OH)D level (16.41 ng/ml) of the control group was still below the reference value. Logistic regression analysis showed that 25(OH)D was a significant predictor of DVT. Weight, height and body mass index, which all presented interaction, were significant in the logistic regression analysis but not in individual analyses.

**CONCLUSION:**

The serum vitamin D levels of DVT patients were lower than those of controls. If the results obtained from our study are supported by further large-scale randomized controlled trials, vitamin D replacement may be brought into the agenda for protection against DVT.

## INTRODUCTION

Vitamin D is a fat-soluble vitamin that is synthesized non-enzymatically in the skin and metabolized in the liver and kidneys. It arranges the immune response of the body, acts as a steroid hormone and plays a crucial role in mineral homeostasis and skeletal health.^1^ Serum vitamin D levels in the range 30-60 ng/ml (75-150 nmol/l) are considered normal. Deficiency of vitamin D is associated with a variety of bone disorders (rickets, osteoporosis or osteomalacia), skin diseases and autoimmune disorders.^1^^,^^2^ It also causes increased risk of cardiovascular diseases such as myocardial infarction (MI), heart failure and coronary artery disease.^2^^–^^5^ Furthermore, deficiency of vitamin D has been reported in arterial diseases, including aortic aneurysm, peripheral arterial disease, arterial calcification and hypertension.^6^^–^^9^

Deep vein thrombosis (DVT) is characterized by thrombus formation, usually in the lower-extremity deep venous system, which causes obstruction or occlusion of blood flow in veins. It is considered to be the third most common cardiovascular disease, after ischemic heart diseases and cerebrovascular events.^10^ Although the medical and interventional treatment options for deep vein thrombosis have improved nowadays, it continues to pose a serious problem, especially in cases with inadequate treatment. It can lead to pulmonary thromboembolism, venous gangrene, chronic venous insufficiency and post-thrombotic syndrome.^11^ The most well-known factors in the etiology of lower-extremity deep vein thrombosis are genetic predisposition, malignancy, history of surgical operation, immobilization, trauma, bone fractures, long journeys and oral contraceptive use.^12^ Nevertheless, there may also be other factors that play a role in the etiopathogenesis of deep vein thrombosis.

## OBJECTIVE

There are very few studies in the existing literature on the topic of the association between vitamin D levels and lower-extremity deep vein thrombosis. Therefore, we designed this study to investigate whether deficiency of vitamin D is associated with lower-extremity deep vein thrombosis.

## METHODS

### Ethical considerations

Ethical approval regarding this study was obtained from the institutional ethics committee (decision: 4/17; date: March 28, 2018). All the participants in this study were only included after written informed consent had been obtained from them. All procedures performed in this study were compatible with the ethical standards of the institutional research committee and with those of the Declaration of Helsinki and its comparable ethical standards.

### Study design and participants

This was a case-control study that was conducted in the cardiovascular surgery and family medicine departments of our hospital between January 2018 and December 2018. A total of 280 participants were included in the study and were divided equally into two groups: the study group (n = 140) and the control group (n = 140).

The study group consisted of patients who had been admitted to the cardiovascular surgery department and had been diagnosed as presenting lower-extremity deep vein thrombosis. The patients with this condition had had signs and symptoms such as pain and swelling in the leg at the time of admission. After the initial examination on patients whose clinical condition had given rise to a suspicion of deep vein thrombosis, lower-extremity venous duplex ultrasonography was routinely carried out in order to confirm the final diagnosis. All the patients with lower-extremity deep vein thrombosis received anticoagulant therapy consisting of warfarin sodium, low molecular weight heparin or new oral anticoagulant agents such as rivaroxaban.

On the other hand, individuals who were admitted to the family medicine department for a routine check-up and consented to participate in the study were enrolled in the control group. All the participants’ demographic and basic clinical characteristics, their comorbid diseases and some laboratory parameters (including vitamin D levels) were noted and then compared between the groups. Findings of any significant variations between the study and control groups, especially with regard to vitamin D levels, were examined in the study. Participants with a family history of venous thromboembolism (VTE) and those who had previously been diagnosed with this condition, individuals who had suffered liver or kidney failure, pregnant women, individuals who had undergone major surgery or trauma in the previous three months and those who had been receiving vitamin D supplementation or hormone replacement therapy over the previous two years were excluded from the study.

### Determination of vitamin D levels

Venous blood samples were taken from the subjects enrolled in the study. The samples were placed in sterile standard tubes. Plasma levels of 25-hydroxyvitamin D (25(OH)D), which is a marker for vitamin D status, were measured by means of chemiluminescence immunoassay. All the samples were analyzed within one hour of collection. The levels of 25(OH)D were categorized into three groups: i) Sufficient group, 25(OH)D > 30 ng/ml; ii) Insufficient group, 25(OH)D = 20-30 ng/ml; and iii) Deficient group, 25(OH)D < 20 ng/ml.

### Statistical analysis

The normality of the variables was evaluated using the Anderson-Darling test. Descriptive statistics were acquired. Data were expressed as number (%) or median (minimum-maximum). Continuous variables were compared using the Mann-Whitney U-test. Categorical data (two-way tables) were evaluated using the chi-square test. Receiver operating characteristic (ROC) curve analysis was used to determine the cutoff values of 25-hydroxyvitamin D for deep vein thrombosis from the area under the curve (AUC). The ROC curve analysis was performed using the “OptimalCutpoints” library (version 1.1-4), which was described by López-Ratón et al. for the R software (version 3.4).^13^

Multiple explanatory variable logistic regression analysis was then conducted. The initial model was fitted with inclusion of all significant independent variables. Following this, a backward-elimination approach in a multiple explanatory variable logistic regression model was conducted to evaluate the model for potential confounding effects. In this model, the factors/covariates were taken away one at a time, starting with the factor/covariate that had the largest P value, until all remaining factors had a two-tailed P-value < 0.05. The goodness of fit was tested using the Hosmer-Lemeshow test. The single and multiple explanatory variable logistic regression analysis methods were used. In the single explanatory variable logistic regression analysis, we estimated the odds ratios (OR) with 95% confidence intervals (CI) for deep vein thrombosis, for each study variable, and the significance level of each factor/covariate was determined. The analyses were performed in R (R Core Team, 2014).^14^

## RESULTS

The study group consisted of 71 males and 69 females, while the control group consisted of 67 males and 73 females. The mean age of the patients with deep vein thrombosis was found to be 58.36 ± 16.36 years, while it was 57.95 ± 16.01 years in the control group (P = 0.814). The 25(OH)D levels of the patients with deep vein thrombosis were found to be significantly lower than those of the control group: median 9.14 (minimum-maximum: 4.2-82.5) versus 16.41 (4.2-70.7) ng/ml; P < 0.001. Female patients in the study group had significantly lower 25(OH)D levels than those in the control group: median 7.34 (minimum-maximum: 4.20-79.80) versus 12.57 (4.20-70.71) ng/ml; P = 0.002 (Table 1). Similarly, male patients in the study group had significantly lower 25(OH)D levels than those in the control group: median 10.59 (minimum-maximum: 4.20-56.21) versus 22.12 (4.20-82.51) ng/ml; P < 0.001.

**Table 1:** Demographics, clinical characteristics and vitamin D status of the participants

Characteristic	DVT group (n = 140)	Control group (n = 140)	P-value
Age (years)	60 (20-91)	60 (20-90)	0.814
Female gender, n (%)	69 (49.3%)	73 (52.1%)	0.720
Height (cm)	168 (145-190)	167 (138-191)	0.079
Weight (kg)	77 (55-120)	75 (40-122)	0.056
BMI (kg/m^2^)	27.3 (19.5-44.1)	27.0 (18.2-39.4)	0.335
Obesity, n (%)	35 (25.0%)	32 (22.8%)	0.779
Diabetes mellitus, n (%)	17 (12.1%)	23 (16.4%)	0.393
Hypertension, n (%)	22 (15.7%)	19 (13.6%)	0.735
Hyperlipidemia, n (%)	12 (8.6%)	12 (8.6%)	1.000
Smoking, n (%)	36 (25.7%)	36 (25.7%)	1.000
25(OH)D level (ng/ml)	**9.15 (4.20-82.51)**	**16.41 (4.20-70.77)**	**< 0.001**

DVT = deep vein thrombosis; BMI = body mass index; 25(OH)D = 25-hydroxyvitamin D.

Data are expressed as median (minimum-maximum) for continuous variables or number (%) for categorical variables.

Regarding cutoff values, significant differences were found between the study and control groups. Higher number of patients in the deep vein thrombosis group were found to have vitamin D deficiency, in comparison with the healthy participants, whereas the control group was found to have a greater number of participants with sufficient vitamin D (Tables 1 and 2) (P < 0.001). As shown in Table 3, the 25(OH)D level in the deep vein thrombosis patients was mostly deficient, compared with the sufficient and insufficient subgroups (P < 0.001).

**Table 2:** Assessment of vitamin D measurements

25(OH)D level	DVT group (n = 140)	Control group (n = 140)	Total (n = 280)
Sufficient (25(OH)D > 30 ng/ml)	9 (6.4%)	29 (20.7%)	38 (13.6%)
Insufficient (25(OH)D = 20-30 ng/ml)	14 (10.0%)	36 (25.7%)	50 (17.9%)
Deficient (25(OH)D < 20 ng/ml)	**117 (83.6%)**	**75 (53.6%)**	**192 (68.6%)**

DVT = deep vein thrombosis; 25(OH)D = 25-hydroxyvitamin D.

Data are expressed as number (%) for categorical variables.

**Table 3:** Demographics, clinical characteristics and vitamin D status of the participants in the deep vein thrombosis (DVT) subgroups based on 25-hydroxyvitamin D (25(OH)D) status

Characteristic	Sufficient (n = 38)	Insufficient (n = 50)	Deficient (n = 192)	P-value
Age (years)	59 (22-84)	63 (38-90)	61 (20-91)	0.500
Female gender, n (%)	20 (52.6%)	18 (36.0%)	104 (54.2%)	0.071
Height (cm)	167 (145-187)	165 (145-180)	168 (138-191)	0.227
Weight (kg)	77 (48-116)	75 (44-114)	76 (40-122)	0.916
BMI (kg/m^2^)	28.2 (18.2-37.1)	27.6 (19.3-38.8)	27.0 (19.5-44.1)	0.281
Obesity, n (%)	11 (28.9%)	17 (34.0%)	39 (20.3%)	0.096
Diabetes mellitus, n (%)	3 (7.9%)	8 (16.0%)	29 (15.1%)	0.474
Hypertension, n (%)	4 (10.5%)	5 (10.0%)	32 (16.7%)	0.367
Hyperlipidemia, n (%)	1 (2.6%)	8 (16.0%)	15 (7.8%)	0.500
Smoking, n (%)	12 (31.6%)	8 (16.0%)	52 (27.1%)	0.188
25(OH)D level, n (%)	**9 (23.7%)**	**14 (28.0%)**	**117 (60.9%)**	**< 0.001**

BMI = body mass index; 25(OH)D = 25-hydroxyvitamin D.

Data are expressed as median (minimum-maximum) for continuous variables or number (%) for categorical variables.

**Figure 1:**
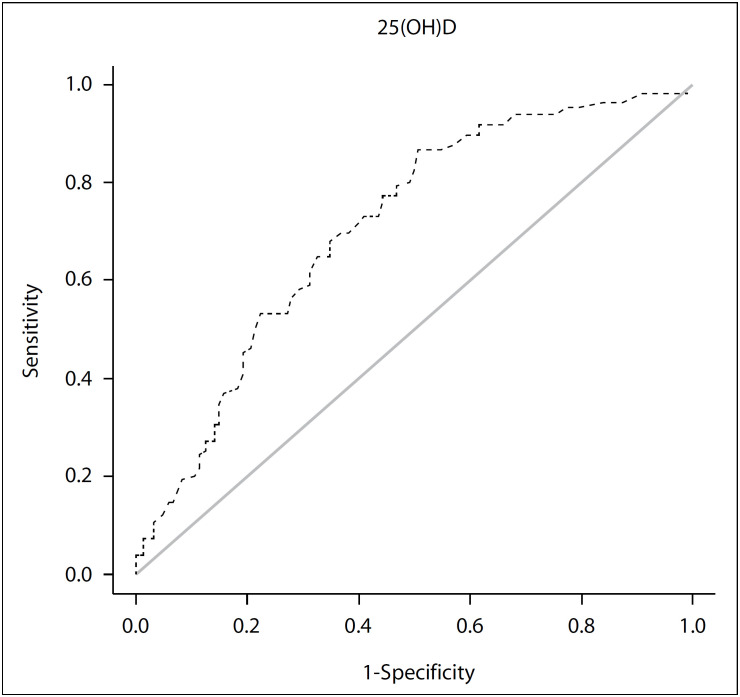
Receiver operating characteristic (ROC) curve showing the diagnostic value of serum 25-hydroxyvitamin D (25(OH)D) for predicting deep vein thrombosis (DVT).

The results from the logistic regression analysis that was performed in order to determine independent predictors of deep vein thrombosis are presented in Table 4. According to this logistic regression analysis, 25(OH)D was shown to be a significant predictor of deep vein thrombosis. In addition, body mass index, weight and height, which all presented interaction, were also significant in the logistic regression analysis, but not in individual analyses. 25(OH)D was found to be a significant variable in ROC analysis (Figure 1).

**Table 4:** Logistic regression analysis to determine independent predictors for deep vein thrombosis (DVT)

	P-value	Odds ratio (95% CI)
Age	0.440	1.0063 (0.9904, 1.0225)
Gender	0.442	0.8168 (0.4875, 1.3685)
Height	**0.010**	1.2718 (1.0472, 1.5445)
Weight	**0.022**	0.7976 (0.6501, 0.9786)
BMI	**0.015**	1.9402 (1.0988, 3.4258)
Obesity	0.684	1.2052 (0.4897, 2.2962)
Diabetes mellitus	0.126	0.5800 (0.2871, 1.1719)
Hypertension	0.680	1.1612 (0.5701, 2.3649)
Hyperlipidemia	0.734	0.8548 (0.3466, 2.1079)
Smoking	0.953	0.9830 (0.5524,1.7491)
25(OH)D	**< 0.001**	**0.9501 (0.9272, 0.9536)**

CI = confidence interval; BMI = body mass index; 25(OH)D = 25-hydroxyvitamin D.

Consequently, serum 25(OH)D values are thought to have diagnostic value for predicting deep vein thrombosis, but height, weight and body mass index have no combined diagnostic value for predicting deep vein thrombosis.

## DISCUSSION

In the current study, we compared the 25(OH)D levels of healthy participants with those of patients diagnosed with deep vein thrombosis. It was found that deep vein thrombosis was more common among patients who had significantly decreased 25(OH)D levels and vitamin D deficiency.

Vitamin D deficiency is a major health problem affecting many people worldwide. The frequency of vitamin D deficiency in healthy individuals in Middle Eastern countries has been reported to be 30-50%.^15^^–^^16^ Although there is no consensus on the definition of vitamin D deficiency, the majority of authors have reported that 25(OH)D levels below 20 ng/ml can be considered to constitute vitamin D deficiency. It has been indicated that for individuals to remain healthy, their serum 25(OH)D levels need to be continually above 30 ng/ml.^1^^,^^17^

Vitamin D has antithrombotic properties, and the major mechanisms reported for these properties include upregulation of thrombomodulin and downregulation of tissue factor. This also increases the levels of interleukin 10 (IL-10), which is an anti-inflammatory cytokine.^15^ It has been reported that greater exposure to ultraviolet B light improves vitamin D status, which positively affects anticoagulant properties and cytokine profile.^18^ Since vitamin D levels have been shown to be inversely related to plasminogen activator inhibitor-1 (PAI-1) levels, vitamin D is also associated with fibrinolytic activity and vascular endothelial integrity.^19^ Therefore, it is possible that vitamin D has positive effects on both thrombosis and fibrinolysis. High prevalence of vitamin D deficiency was found in all seasons among 478 subjects diagnosed with acute myocardial infarction (MI), although the deficiency was lower in summer than in winter.^4^

The major risk factors for myocardial infarction consist of atherosclerosis and formation of thrombi and emboli. Inflammation plays an essential role in the basis for risk factor pathogenesis, and vitamin D deficiency increases inflammation. Thus, vitamin D supplementation may reduce these factors.^20^ Vitamin D also decreases endoplasmic reticulum stress and oxidative stress of endothelial cells, thereby reducing the risk of atherosclerosis and thrombosis.^21^

Only a very limited number of studies in the existing literature have investigated the relationship between vitamin D levels and venous thromboembolism (VTE). Moreover, to the best of our knowledge, there is only one study in the literature that has examined the association between vitamin D and lower-extremity deep vein thrombosis. Khademvatani et al. studied the relationship between vitamin D status and idiopathic lower-extremity deep vein thrombosis in a case-control study on 82 deep vein thrombosis patients. They reported that low levels of 25(OH)D were related to occurrences of idiopathic lower-extremity deep vein thrombosis.^22^ In another study, Wu et al. investigated whether low serum 25(OH)D levels were related to increased incidence of deep vein thrombosis in patients with ischemic stroke. They concluded that low levels of 25(OH)D were independent predictors for deep vein thrombosis in patients with ischemic stroke.^23^ Moreover, these authors also indicated that the findings of their study revealed the critical role played by 25(OH)D in the pathogenesis of deep vein thrombosis. On the other hand, the Tromsø Study examined whether high 25(OH)D levels were related to decreased risk of VTE in a prospective population-based study with 6,021 participants. The study revealed that serum vitamin D level was not related to future risk of deep vein thrombosis.^24^

However, in a large-scale observational study including 18,791 participants, it was observed that the risk of deep vein thrombosis became higher with decreasing terciles of seasonally arranged plasma 25(OH)D concentrations. It was concluded that randomized controlled trials were required in order to test the question of causality and whether vitamin D supplementation was essential in the overall population, or only in selected patient groups, to reduce the risk of deep vein thrombosis.^25^

The most important limitations of the present study were the retrospective nature of its data collection, its single-centered design and its limited data evaluation.

## CONCLUSION

Our study demonstrated that the serum vitamin D levels of patients with lower-extremity deep vein thrombosis were lower than those of the control subjects. This result suggests that vitamin D deficiency may play a role in the etiopathogenesis of deep vein thrombosis. If the results obtained from our study are supported by further large-scale randomized controlled trials, vitamin D replacement may be brought into the agenda relating to protection against deep vein thrombosis.
